# Comparison of OX40 expression in patients with multiple sclerosis and neuromyelitis optica as an approach to diagnosis

**DOI:** 10.1186/s13223-023-00772-9

**Published:** 2023-03-10

**Authors:** Mostafa Manian, Morteza Motallebnezhad, Reza Nedaeinia, Rasoul Salehi, Leila Khani, Gordon A. Ferns, Mir Hadi Jazayeri

**Affiliations:** 1 Child Growth and Development Research Center, Research Institute for Primordial Prevention of Non-Communicable Disease, Isfahan University of Medical Sciences, Isfahan, Iran; 2 Department of Immunology, School of Medicine, Iran University of Medical Sciences, Shahid Hemmat Highway, P.O Box: 14665-354, Tehran, 1449614535 Iran; 3 Pediatric Inherited Diseases Research Center, Research Institute for Primordial Prevention of Non-Communicable Disease, Isfahan University of Medical Sciences, Isfahan, Iran; 4 Department of Genetics and Molecular Biology, School of Medicine, Isfahan University of Medical Sciences, Isfahan, Iran; 5 Division of Medical Education, Brighton and Sussex Medical School, Falmer, Brighton, Sussex, BN1 9PH UK; 6 Immunology Research Center, Iran University of Medical Sciences, Tehran, Iran; 7 Laboratory of Transcriptional Regulation, Institute of Medical Biology, Polish Academy of Science, Lodz, Poland; 8 Bio-Med-Chem Doctoral School of the University of Lodz, Lodz Institutes of the Polish Academy of Sciences, Lodz, Poland

**Keywords:** CD134, Multiple sclerosis, Neuromyelitis optica, OX40, T cell

## Abstract

**Background:**

Previous studies have shown that CD134 (OX40) co-stimulation is involved in the pathogenesis of experimental autoimmune encephalomyelitis (EAE) models and the antigen is expressed within multiple sclerosis lesions in humans. OX40 (CD134) is thought to be a secondary co-stimulatory immune checkpoint molecule that is expressed by T cells. This study aimed to evaluate the mRNA expression of OX40 and its serum levels in the peripheral blood of patients with Multiple Sclerosis (MS) or Neuromyelitis Optica (NMO).

**Methods:**

Patients with MS (n = 60), NMO (n = 20), and 20 healthy subjects were recruited from Sina Hospital, Tehran, Iran. The diagnoses were confirmed by a specialist in clinical neurology. Peripheral venous blood was obtained from all subjects, and mRNA quantification of OX40 was conducted using real-time PCR. Serum samples were also obtained and the concentration of OX40 was determined using an enzyme-linked immunosorbent assay (ELISA).

**Results:**

There was a significant correlation between the mRNA expression and serum levels of OX40 and disability as assessed using the expanded disability status scale (EDSS) in the patients with MS, but not in the patients with NMO. Expression of OX40 mRNA was significantly higher in the peripheral blood of MS patients compared to healthy individuals and NMO patients (*P < 0.05). In addition, serum OX40 concentrations were also significantly higher in patients with MS patients compared with healthy subjects (9.08 ± 2.48 vs. 1.49 ± 0.54 ng/ml; P = 0.041).

**Conclusions:**

It appears that an increased expression of OX40 may be associated with the hyperactivation of T cells in patients with MS, and this may play a role in the pathogenesis of the disease.

## Introduction

MS is an autoimmune disorder of the central nervous system (CNS), characterized by several neuronal and spinal cord symptoms mediated by inflammation, demyelination, and axonal damage [1–3]. Among the histological hallmarks of early MS lesions is demyelination, resulting in destruction of myelin and the related axons. The axonal loss occurring during MS is responsible for permanent disability. Immune complications have been widely implicated in the pathogenesis of MS [4]. Abnormalities of the T cell lineage have been reported to occur with a divergence of helper T (Th)1 and Th17 cells and dysfunction of regulatory T (Treg) in MS patients, leading to an inflammatory response [5]. Moreover, aberrant production of cytokines and matrix metalloproteinases (MMPs) is involved in the disruption of the blood brain barrier (BBB) in MS [6]. Disruption of BBB increases the expression of adhesion molecules on blood vessels and leads to the migration of T cells to the CNS, where the further activation of these cells eventuates in the stimulation of damaging inflammatory cascade of events. In addition to CD4^+^ T cell activation and differentiation to pathogenic Th1 and Th17 cells, CD8^+^ T cells may play an important role during inflammation of the CNS [7]. Immune cell co-stimulatory pathways have been shown to facilitate T cell activation. Furthermore, there is growing evidence indicating that co-stimulatory pathways affect the function of other inflammatory cells both in the CNS and periphery, possibly modulating the function of glial and neural cells [8–10]. Tumor necrosis factor receptor superfamily member 4 (TNFRSF4), also known as CD134 and OX40, belongs to the TNFR-superfamily. OX40 is considered to be a secondary co-stimulatory immune checkpoint molecule that is expressed after T cell activation [11]. OX40 ligand (OX40L) is also a member of the TNF family which is not expressed on resting antigen-presenting cells (APCs); rather it is expressed following the activation of B cells [11], dendritic cells [12], and endothelial cells [13]. In the absence of CD28, OX40 binding to OX40L results in the interleukin (IL)-2 production and T cell proliferation [14]. It has been reported that treatment with natalizumab reduces OX40^+^ CD26^+^ CD4^+^ T cells in the periphery of MS patients [15]. Furthermore, studies in animal models of MS, namely EAE, has shown the involvement of OX40-OX40L interactions in the complications of the CNS [16]. OX40L-deficient mice were reported to have a milder course of EAE and reduced proliferation of T cell and IL-2, IL-6, and interferon (IFN)-γ production. Moreover, OX40L-transgenic mice presented more severe clinical course of EAE [17]. In inflammatory conditions such as inflammatory bowel disease, the CNS in mice with EAE were detected as having high expressions of OX40 [18] and both CD28 and OX40 signals are necessary for the optimal development of memory CD4 T cells [19].

With respect to the potential involvement of OX40-OX40L interactions in the activation of T cells, it has been suggested as a potential therapeutic target for autoimmunity; moreover, effector T cells are major players of MS etiopathogenesis. NMO is an inflammatory disease resulting from demyelinating of the CNS with involvement of the optic nerves and spinal cord which is mediated in most cases by antibodies. Anti-AQP4-IgG is used as an important diagnostic criterion for NMO disease [20]. Some NMO patients are seronegative and may have a heterogeneous spectrum of NMO or similar disease like MS [21].

In both MS and NMO, the nervous system is attacked by the immune system, but CNS is involved in different inflammation sites for each disease [22]. In more than 70% of NMO specific antibodies—the NMO-IgG or anti-AQP4 antibody—is positive, although there are clinically typical cases confirmed in terms of imaging characteristics with negative antibodies in many cases [23, 24]. In MS, the immune system attacks the entire CNS and the myelin, which causes the symptoms of MS, while in NMO, the immune system attacks only the brain and spinal cord [23]. The symptoms of MS relapse are less severe than NMO, especially in early stages of the disease and NMO does not have a progressive disease course similar to MS [25].

The term NMO spectrum disorder (NMOSD) is used to include limited phenotypes such as recurrent optic neuritis or myelitis and NMO (including both optic neuritis and myelitis) [26]. Similar symptoms to NMO can also be seen in relapsing–remitting multiple sclerosis (e.g. attacks of myelitis and optic neuritis) [26]. There is a need for more specific diagnostic modalities and improved diagnostic assays and methods. This study aimed to improve differential diagnosis of MS and NMO disease and to provide a better understanding of the basic immunology of NMO and NMOSD [21]. As NMO and MS diseases integrated during a long time the same nosologic complex, their distinction and identification of other differential parameters may improve approaches related to clinical evolution, treatment and prognosis [27].

We aimed to evaluate the mRNA expression and serum level of OX40 in the peripheral blood of MS patients and compared with NMO patients, and healthy controls.

## Methods

### Study design

This study was performed on 60 patients with relapsing–remitting MS (RRMS) referred to Sina hospital, Iran, during 2018, 20 NMO patients, and confirmed by clinician neurologist and 20 healthy controls. Subjects with chronic inflammatory and autoimmune disease and those under drug therapy were excluded from the study. Healthy individuals were also excluded if they had autoimmune diseases and a familial history of auto-inflammatory conditions. MS and NMO patients and healthy controls were age- and sex-matched. MS patients were diagnosed as having MS based upon the McDonald criteria [28, 29], and the disability score of patients was calculated using the expanded disability status scale (EDSS) as described previously [30]. EDSS is a method of calculating disability in multiple sclerosis and screening changes in the level of disability over time and provides effective and reliable assessment at every stage of the disease. Scoring is based on an examination by a neurologist and mainly based on the evaluation of functional systems [31]. Moreover, the NMO patients were diagnosed based on the international panel for NMO Diagnosis (IPND) criteria [32], and the disability score of patients was further determined by EDSS score. MS subjects fell under the RRMS category, and the selected patients in the remitting state were those with no immunomodulatory drugs received for at least 3 months prior to sampling. This study was approved by the Human Research Ethics Committee of the Iran University of Medical Sciences, Tehran, Iran, and informed consent forms were obtained by all MS, NMO, and healthy individuals. Using venipuncture, 2 ml of venous blood from all participants was collected in EDTA tubes to isolate mRNA and serum.

### mRNA extraction and cDNA synthesis

In order to extract mRNA from peripheral blood, the RNeasy Mini Kit (Qiagen, Germany) was exerted according to the manufacturer’s instructions. The yield and purity of the extracted RNAs were determined using a NanoDrop spectrophotometer at 260/280 nm (NanoDrop ND-2000C Spectrophotometer, Thermo Fisher Scientific, USA). Next, the first strand complementary DNA (cDNA) was synthesized using the Revert Aid First cDNA synthesis (K1632, Fermentas, Thermo Fisher Scientific) according to the manufacturer’s protocol. Reverse transcription was performed with the final volume of 20 µl per tube. Briefly, 4 µl of isolated RNA (30 µg) was mixed with 1 µl of random hexamer primer and 7 µl of distilled RNase-free H_2_O and incubation was conducted at 65 °C for 5 min. The microtubes were cooled on ice and a mixture of 4 μl of reaction buffer, 1 μl of RNase inhibitor, 2 μl of dNTP mixture and 1 μl of reverse transcriptase was added to each sample. Five-minute incubation at 25 °C followed by 60-min incubation at 42 °C was conducted immediately after sample collection. Then the reaction was stopped by heating for 5 min at 70 °C.

### Real-time PCR quantification

Specific primer sets for OX40 mRNA and Glyceraldehyde-3-Phosphate Dehydrogenase (GAPDH), as the housekeeping gene, were designed using the primer designing tool, NCBI, and NIH (https://www.ncbi.nlm.nih.gov/tools/primer-blast/). The primer sequences for the real-time quantification of the OX40 mRNA expression were the forward primer of 5′-TGGTGTAACCTCAGAAGTG-3′ and the reverse primer of 5′-GTCAACTCCAGGCTTGTA-3′; regarding GAPDH, it was the forward primer of 5′-AAGCTCATTTCCTGGTATG-3′ and the reverse primer of 5′-CTTCCTCTTGTGCTCTTG-3′. The NCBI Primer Blast Tool (http://www.ncbi.nlm.nih.gov/tools/primer-blast/) was used to determine the specificity and accuracy of primers. To conduct the real-time gene expression, the Chromo4 (BioRad, USA) PCR machine and StepOne plus PCR system were utilized. The reaction mixture included 10 μl of SYBR Green PCR master mix and 8 μl of template cDNA, 0.6 μl of primers (0.3 μl of each), and 1.4 μl of RNase-free H_2_O to a final volume of 20 μl. The thermal cycles of the thermocycler were as following: 40 cycles of 50 °C for 30 s, 95 °C for 5 s and then 60 °C for 45 s. To normalize the mRNA expression level of the target gene (OX40), the expression level of the housekeeping gene GAPDH was measured in each sample. A cycle threshold (Ct) was calculated for each PCR reaction and the relative expression level of OX40 mRNA was determined as described by Schmittgen and Livak (relative expression = 2^−ΔΔCT^) [33].

### Soluble serum OX40 concentration

To determine the soluble serum OX40 level, sera were prepared from the peripheral blood of 60 MS patients, 20 NMO subjects, and 20 healthy controls. OX40 serum level was evaluated via ELISA using a commercial kit (Human sCD134 (OX40) ELISA Kit, OriGene Technologies, Inc., Rockville, MD, USA).

### Statistical analysis

Data analysis and graph plotting were carried out using SPSS software v. 21 (SPSS, Chicago, IL, USA) and GraphPad Prism v. 7.00 software (GraphPad Software, Inc., San Diego, CA, USA, www.graphpad.com), respectively. The normality of data distribution was specified by the Kolmogorov–Smirnov test. The Kruskal–Wallis nonparametric one-way analysis of variance was applied to compare the three groups in MS and NMO patients as well as healthy controls. Correlation analysis was performed by Spearman’s rho tests. Data was shown as mean ± standard error of the mean (SEM) and statistical significance level was set at *P-*value *< 0.05.

## Results

### Baseline and clinical characteristics of study subjects

Table 1 shows the characteristics of the study participants. In the MS, NMO, and healthy control groups, the male/female ratio was 14 (23%)/46 (77%), 5 (25%)/15 (75%), and 6 (30%)/14 (70%), respectively. The patients and healthy controls were matched with respect to gender distribution. The mean age of the groups with MS, NMO, and healthy individuals was 33.7 ± 9.6, 37.2 ± 7.4, and 30.36 ± 8.8 years respectively. The age difference was not statistically different. The EDSS value of MS and NMO subjects was 1.5 ± 1.88 and 1.55 ± 2.17, respectively.

**Table 1:** Baseline characteristics and demographic data of study participants

Characteristic	MS (n = 60)	NMO (n = 20)	HC (n = 20)	*P* value
Gender (male/female)	14 (23%)/46 (77%)	5 (25%)/15 (75%)	6 (30%)/14 (70%)	> 0.05
Age	33.7 ± 9.6	37.2 ± 7.4	30.36 ± 8.8	> 0.05
EDSS	1.5 ± 1.88	1.55 ± 2.17	–	> 0.05

*MS* multiple sclerosis, *NMO* neuromyelitis optica, *HS* healthy subjects, *EDSS* expanded disability status scale

### Relative mRNA expression of OX40

**Fig. 1:**
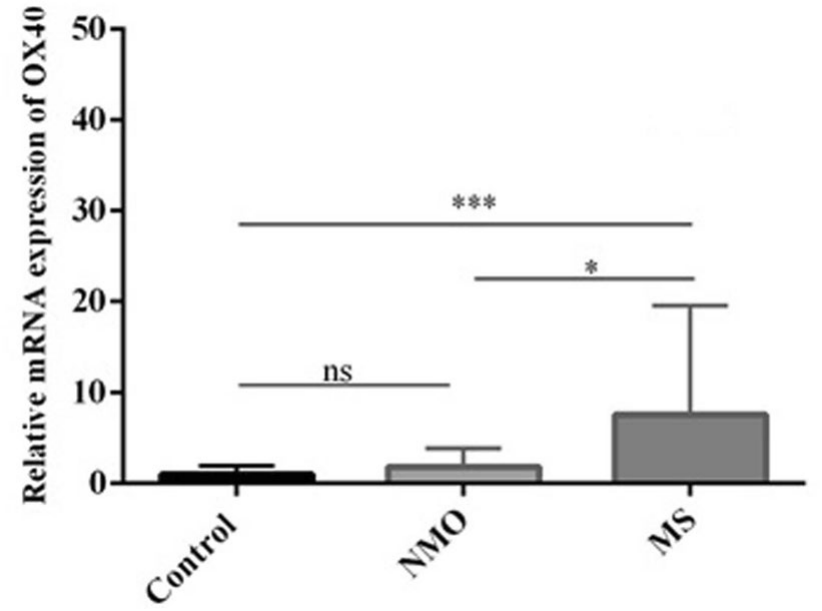
Bar graph illustrates the relative mRNA expression of the OX40 gene in the peripheral blood of MS and NMO patients as well as healthy controls

As shown in Fig. 1, the OX40 mRNA expression in the peripheral blood of MS patients was significantly higher in comparison to that of NMO patients (*P* = 0.0125) and healthy subjects (*P* = 0.0006). Nonetheless, although NMO subjects indicated upregulated levels of OX40 mRNA in peripheral blood in comparison to that of healthy subjects, no statistically significant difference was detected (*P* = 0.383).

### Serum level of OX40

**Fig. 2:**
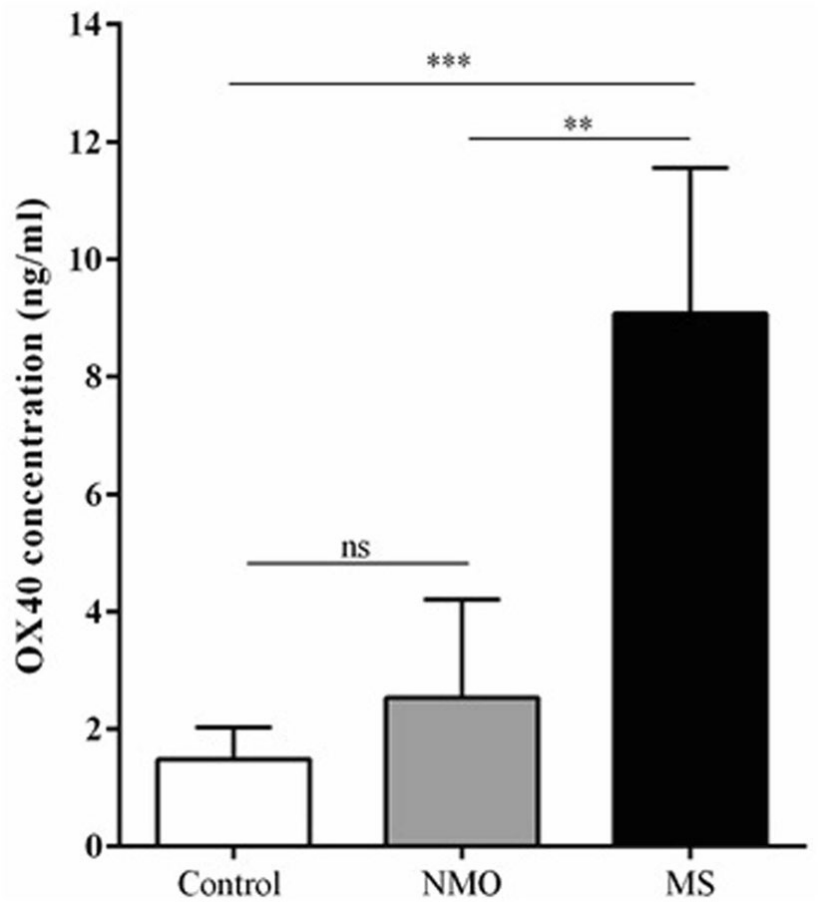
Bar graph illustrates the serum level of OX40 in MS and NMO patients as well as healthy controls

The serum level of OX40 was significantly higher in the MS patients compared to healthy subjects (9.08 ± 2.48 vs. 1.49 ± 0.54 ng/ml; *P* = 0.041). Also, the difference between MS patients and NMO subjects was—statistically significant (9.08 ± 2.48 vs. 2.54 ± 1.67 ng/ml; *P* = 0.034). There was no statistically significant difference in the serum level of OX40 in NMO patients in comparison to healthy subjects (2.54 ± 1.67 vs. 1.49 ± 0.54 ng/ml; *P* = 0.11; Fig. 2).

### Correlation analyses

**Fig. 3:**
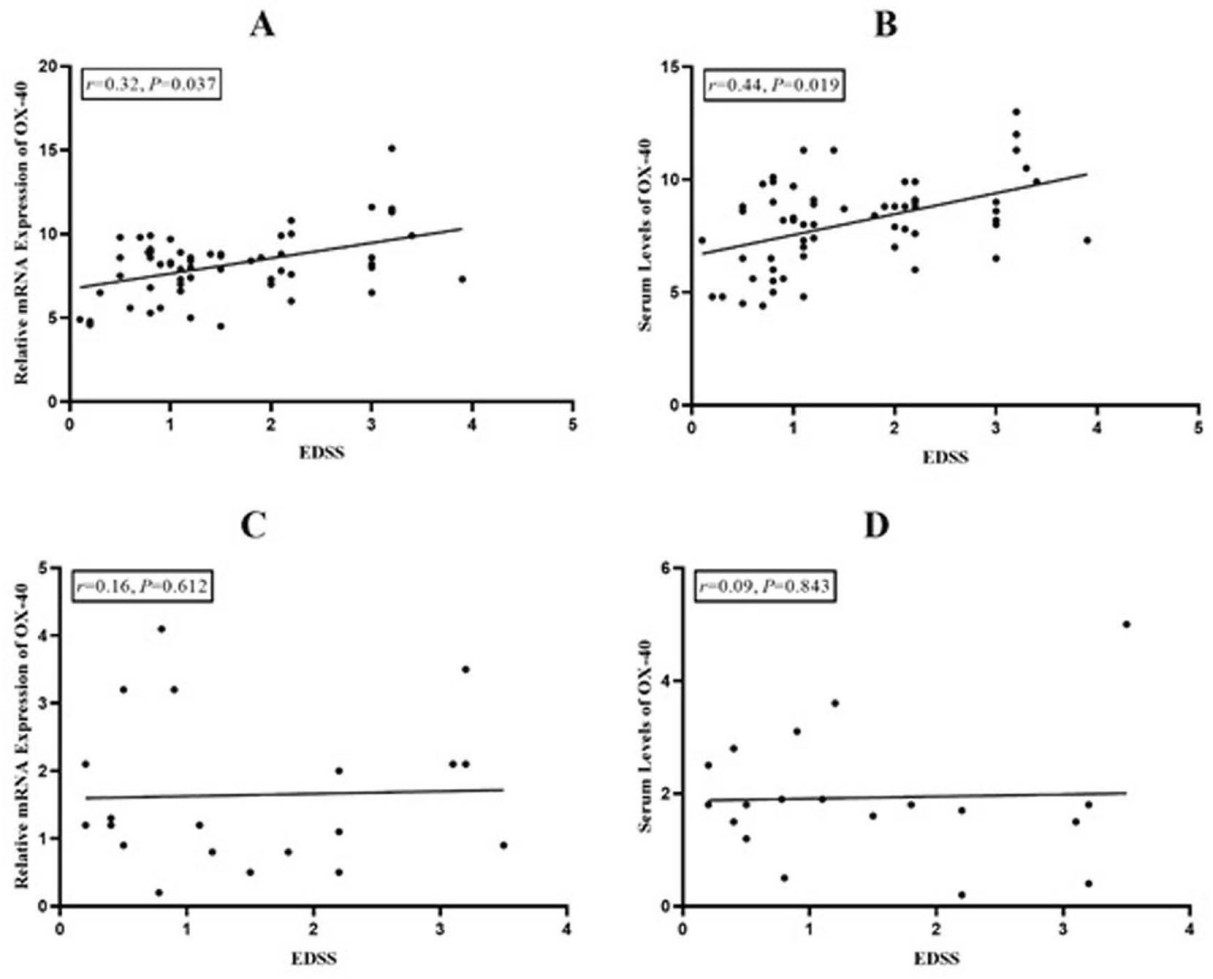
Dot plots and correlation analysis for the mRNA expression and serum levels of OX-40 with the EDSS score in MS and NMO patients. **A** Correlation of EDSS with relative mRNA expression of OX-40 in MS patients. **B** Correlation of EDSS with serum levels of OX-40 in MS patients. **C** Correlation of EDSS with relative mRNA expression of OX-40 in NMO patients. **D** Correlation of EDSS with serum levels of OX-40 in NMO patients

A statistically significant correlation was observed between the mRNA expression of OX40 and EDSS scores in MS patients (*r* = 0.32, *P* = 0.037; Fig. 3A). Furthermore, a significant correlation was found between the serum levels of OX40 and EDSS value in the MS subjects (*r* = 0.44, *P* = 0.019; Fig. 3B). However, no significant correlation was found between the mRNA expression and serum levels of OX40 and EDSS concerning NMO subjects.

## Discussion

We aimed to evaluate the mRNA and serum levels of OX40 in the peripheral blood of MS patients, and compare them with that of NMO patients and healthy controls. The results indicated increased expressions and serum levels of OX40 in the peripheral blood of patients with MS compared to NMO patients and healthy controls. Correlation analysis indicated a significant association between the mRNA expression and serum levels of OX40 in MS patients, but not in NMO subjects. Immune system over-activation has been proposed in the pathogenesis of MS, and several immunotherapeutic approaches have been used to treat the disease by blocking the co-stimulatory molecules involved in the activation of various immune cells during MS [16, 34]. OX40 is expressed on activated T cells and Treg cells, while OX40L is expressed on the APCs, activated T cells, and endothelial and mast cells. The expression of both OX40 and OX40L is upregulated upon antigen presentation, CD28 and CD40L ligation to the corresponding receptors, and IFN-γ signaling. OX40 and OX40L interactions result in T cell proliferation and survival, promoted effector and memory T cell phenotypes, diminished regulatory signaling, enhanced cytokine production, and increased cell mobility [16]. To date, most of the studies related to OX40-OX40L interaction in MS have been conducted on animal models. OX40L-deficient mice showed a milder EAE course, reduced T cell proliferation, and down-modulation of IL-2, IL-6, and IFN-γ production [17]. OX40L expression was observed on the macrophages-microglia present in the CNS of EAE mice [35]. Moreover, in vitro blockade of OX40L on macrophages-microglia resulted in an ex vivo suppression of T cell proliferation, suggesting that OX40-OX40L signaling can play a role in the reactivation of CNS T cells [35]. OX40L expression was further identified on the EAE mice endothelial cells, implying the role of OX40-OX40L signaling in the migration and recruitment of immune cells to CNS [36]. Using a soluble OX40R-Ig molecule to block OX40, resulted in a milder EAE course [35]. In addition, anti-OX40L antibody ameliorated EAE [36], but the treatment led to draining lymph nodes cells showing increased antigen-specific T cell proliferation as well as IFN-γ production. Nonetheless, amelioration of EAE course was correlated with a reduced number of T cells and infiltration of monocytes to the CNS of mice [36]. These findings have implications for the treatment of EAE and possibly MS via blocking OX40-OX40L co-stimulatory signaling. Our analysis further revealed a significant increase in the EDSS value of MS subject (an indicator of disease severity) as the level of OX40 increased, further proposing a plausible therapeutic approach through the inhibition of OX40 or its signaling. On the other hand, natalizumab (a monoclonal antibody against very late antigen (VLA)-4)-treated MS patients had significantly decreased expressions of OX40 on CD4^+^CD26^high^ T cells [15]. We also found an increased expression and serum levels of OX40 in MS patients in comparison to healthy controls. Furthermore, OX40 was implicated in the NMO patients [16]. NMO is defined as an inflammatory demyelinating disease of CNS, in which autoantibodies are developed against aquaporin 4 (AQP4) [37]. It has been reported that active human NMO lesions contain OX40-expressing CD4^+^ T cells. Moreover, expression of Ox40 has reflected the T cell activation and expansion, which was more profound compared to the expression of MS lesions of comparable activity [38]. Our results also demonstrated increased expressions and serum levels of OX40 in NMO patients in comparison to the controls, although the difference was not significant. Nonetheless, NMO patients had significantly lower expressions and serum levels of OX40 compared with MS subjects. Therefore, it seems that OX40–OX40L signaling is more involved in the pathogenesis of MS, and humoral immunity (autoantibodies against AQP4) is predominantly involved in NMO pathogenesis. It has been reported that OX40 is up-regulated at the sites of autoimmunity and correlates with disease severity, especially in neurodegenerative diseases. Fu et al. showed that high levels of peripheral OX40+CD4+ T cells could be detected in systemic disease, but not in local disease, and there was no significant OX40 expression in peripheral blood [39]. Fu et al. also have mentioned that blockade of the OX40–OX40L pathway in vivo generally improves the long‐term autoimmunity, mainly by preventing migration, moderating T cell polarization, altering inflammatory cytokine production, and preventing proliferation of active CD4^+^ T cells [39]. Active CD4+ T cells may play a critical role in MS pathogenesis [40]. Fu et al. has been demonstrated that OX40–OX40L interaction can promote Th1 and Th2 mediated response, Th9 differentiation through the non-canonical NF-kB pathway [39, 41]. Th1 and Th2 cytokine responses especially IFN-γ and IL-2 decreased in OX40L-deficient mice and treatment of wild type mice with anti-OX40L mAb (MGP-34) led to improve clinical symptom of EAE models [17]. Studies in both preclinical and clinical trials have shown that blockade of OX40 or OX40L interaction can improve autoimmunity disease, especially EAE models, through inhibition of auto reactive T cells [16, 39]. In previous study, Nohara et al. using variation dose of neutralizing ox40L mAB (RM134) have shown that significantly reduced the reservoir of OX40-expressing CD4 T cells led to inhibit migration of pathogenic T cells into central nervous system while it had little impact on the proliferation of Th1 cells in the draining lymph nodes (DLN) [36].

Carboni et al. reported that CD134 (OX40) co-stimulation is involved in the pathogenesis of the EAE model and that the antigen is expressed in human multiple sclerosis lesions [42]. They showed that CD134 (also known as OX40) deficiency delayed the onset of clinical symptoms with mild disease progression, eventually leading to a significant reduction in neurological deficits [42].

Drugs that inhibit the OX40 or CD134 pathways may offer an alternative approach to treat patients with multiple sclerosis. EAE injection induced by myelin oligodendrocyte glycoprotein (MOG) in Cd134/mice resulted in less serious clinical symptoms and significantly decreased inflammatory infiltrates in the central nervous system (CNS) [42].

## Conclusions

These studies suggest the involvement of OX40-OX40L signaling in the pathogenesis of MS and provide an insight into the basic immunology of NMO and NMOSD. Our results show the upregulation in the expressions of OX40 mRNA in peripheral blood and increased serum levels of OX40 antigen in MS patients in comparison to healthy controls and NMO patients. In addition, a correlation was detected between the mRNA expressions and serum levels of OX40 and the EDSS of MS patients, but not in NMO subjects, hence the proposition that OX40-OX40L signaling is involved in the pathogenesis of MS, not NMO. It is necessary to conduct further investigations with respect to this signaling pathway on T cells and in MS lesions for a better understanding and designing specific therapeutic tools targeting OX40-OX40L pathway.

## Data Availability

Not applicable.

## Abbreviations

MS: Multiple sclerosis
NMO: Neuromyelitis optica
EDSS: Expanded disability status scale
CNS: Central nervous system
Th: T helper
Treg: Regulatory T cells
BBB: Blood brain barrier
EAE: Experimental autoimmune encephalomyelitis
IFN: Interferon
NMOSD: NMO spectrum disorder
VLA: Very late antigen
MOG: Myelin oligodendrocyte glycoprotein
DLN: Draining lymph nodes
TNFRSF4: Tumor necrosis factor receptor superfamily member 4
